# Supplementary material for: NICEpath: Finding metabolic pathways in large networks through atom-conserving substrate–product pairs
Source: Bioinformatics. 2021 May 18;37(20):3560–8. doi: 10.1093/bioinformatics/btab368 (PMC8545321; doi:10.1093/bioinformatics/btab368)
Supplement: btab368_Supplementary_Data [file btab368_supplementary_data.zip › Supplementary_information.docx]

Supplementary Information

*Note: All supplementary figures can be obtained by running compare_algorithms.py within the NICEpath package on GitHub (*[*https://github.com/EPFL-LCSB/nicepath*](https://github.com/EPFL-LCSB/nicepath)*).*

**Supplementary Figure S1:** The three transformations (i.e., exponential, default, and square root) as a function of the CAR. The vertical black line indicates the CAR cutoff that best predicts KEGG RPAIRs of type “main”.

**Supplementary Figure S2:** Distribution of pathway length within the set of 50 KEGG reference pathways selected for performance evaluation.

**Supplementary Figure S3:** Evaluation of performance in terms of algorithm runtime and rank performance. For each of the 50 reference pathways, the 100 shortest paths have been extracted connecting the source and the end compound of the reference pathway. The rank of the reference pathway within the 100 shortest paths is shown as a metric of performance (e.g., if the rank is 1, the algorithm found the reference pathway first). (**a**) Comparison of runtime between the three proposed transformation operators. (**b**) Comparison of algorithm runtime between the three unweighted networks. (**c**) Performance of the algorithm within the three transformation operators. (**d**) Performance of the algorithm within the three unweighted networks. It should be noted that the k-shortest path algorithm used for the unweighted networks starts the search on the source and the end compound simultaneously, leading to decreased runtimes, while the k-shortest path algorithm for weighted networks starts exploring from the source node only. **Abbreviations:** “Exp”: exponential transformation, “dflt”: default transformation, “sqrt”: square root transformation, “CAR > 0.34”: unweighted version of the original reactant pair network where edges with CAR > 0.34 are kept, and the remaining edges removed, “main RPAIRs”: the RPAIR “main” network from KEGG, “no cofactors”: unweighted version of the original reactant pair network where weights have been removed and cofactors nodes removed. List of cofactors excluded in the “no cofactors” network: NAD+, NADH, H+, NADP+, NADPH, CO2, H2O, Orthophosphate, CoA, Oxygen, Ubiquinone, Ubiquinol, Hydrogen peroxide, FAD, FADH2, Ammonia, Iodide, Tetrahydrobiopterin, Reduced FMN, FMN, Thioredoxin disulfide, Thioredoxin, S-Adenosyl-L-methionine, S-Adenosyl-L-homocysteine, 5-Methyl-5,6,7,8-tetrahydromethanopterin, 5,6,7,8-Tetrahydromethanopterin, GDP, UDP, Diphosphate, S-Adenosylmethioninamine, ATP, ADP, AMP, GTP.

**Supplementary Figure S4**: Performance evaluation of the k-shortest path search algorithm within the three weighted and the three unweighted networks (see Supplementary Figure S3). The distribution of the rank of the reference pathway within the shortest 100 paths is shown for each network. Abbreviations: See caption of Supplementary Figure S3.
